# A Feedback-Based Secure Path Approach for Wireless Sensor Network Data Collection

**DOI:** 10.3390/s101009529

**Published:** 2010-10-22

**Authors:** Yuxin Mao, Guiyi Wei

**Affiliations:** School of Computer and Information Engineering, Zhejiang Gongshang University, Xuezheng Street NO. 18, Hangzhou 310018, Zhejiang, China; E-Mail: weigy@zjgsu.edu.cn

**Keywords:** data collection, feedback, secure path, wireless sensor network

## Abstract

The unattended nature of wireless sensor networks makes them very vulnerable to malicious attacks. Therefore, how to preserve secure data collection is an important issue to wireless sensor networks. In this paper, we propose a novel approach of secure data collection for wireless sensor networks. We explore secret sharing and multipath routing to achieve secure data collection in wireless sensor network with compromised nodes. We present a novel tracing-feedback mechanism, which makes full use of the routing functionality of wireless sensor networks, to improve the quality of data collection. The major advantage of the approach is that the secure paths are constructed as a by-product of data collection. The process of secure routing causes little overhead to the sensor nodes in the network. Compared with existing works, the algorithms of the proposed approach are easy to implement and execute in resource-constrained wireless sensor networks. According to the result of a simulation experiment, the performance of the approach is better than the recent approaches with a similar purpose.

## 1. Introduction

Although intrusion detection is an important issue to wireless sensor networks (WSNs), it is still in its infancy and there are currently only a few of studies in this area. Due to the intrinsic features of WSNs, it is difficult to perform efficient intrusion detection in such a resource-restricted environment [1]. Many intelligent or statistical approaches are too complex for resource-constrained WSNs. In contrast, it is much easier to elude or bypass malicious nodes rather than detect them. One possible solution to such kinds of attacks is to exploit the routing functionality of WSNs. If the locations of the malicious nodes (also called compromised nodes) are known *a priori*, then sensed information can be delivered over paths that circumvent (bypass) malicious nodes, whenever possible. As the existing intrusion detection methods for WSN are still immature, in practice it is difficult to acquire such location information precisely. Therefore, the above idea of delivering information is often implemented in a probabilistic manner. Multipath routing allows the establishment of multiple paths between a single source and single destination node. It is typically proposed in order to increase the reliability of data transmission (*i.e.*, fault tolerance) or to provide load balancing [2]. If the location information of compromised nodes is not known *a priori*, the source node can deliver sensed information by multiple paths to decrease the chance of the information being intercepted.

However, there are still problems with the multipath routing approach. If adversary can selectively compromise sensor nodes, sensed information is intercepted in each fixed routing path even if it can be distributed over different routes. One possible solution to this problem is delivering information randomly through different paths rather than a fixed set of routes [3]. Although an adversary can still intercept part of the information, we can reduce the probability of interception to an acceptable extent using specific methods.

In this paper, we propose a novel approach of secure data collection for WSNs. We explore secret sharing and multipath routing to achieve secure data collection in a WSN with compromised nodes. The remaining of the paper is organized as follows: Section 2 gives an overview of the related works. In Section 3, we present an adaptive multi-path data collection algorithm for WSNs. In Section 4, we propose a feedback-based secure path algorithm for secure data collection in WSNs. We evaluate the approach with a simulation experiment in Section 5 and discuss the simulation results in detail. Section 6 concludes the paper with an outlook to future research directions.

## 2. Related Works

There have been a few on-going research efforts concerning multipath routing for secure data collection presented in literature. For example, the SPREAD algorithm in [4] is used to find multiple most-secure and node-disjoint paths. A modified Dijkstra algorithm is used to find the top-K most secure node-disjoint paths iteratively. The H-SPREAD algorithm [5] improves the SPREAD algorithm by simultaneously accounting for both security and reliability requirements. The work in [6] presents distributed Bound-Control and Lex-Control algorithms, which compute multiple paths respectively. Shu *et al.* in [3] presented an approach for secure data collection by using a (*t*, *n*)-threshold secret sharing algorithm and randomized multipath routes. A packet is broken into shares, which are sent to the sink through randomly generated paths. In their simulation, they use a fixed source node to evaluate the approach in simulation, while we extend their simulation with a collection of source nodes. Nasser and Chen in [7] propose a routing protocol that uses multipath alternately as the path for communicating between two nodes. The protocol defends against some specific attacks like selective forwarding by advertising an attractive route to the destination. Deng *et al.* in [8] propose an intrusion-tolerant routing protocol for WSNs. They try to preserve WSN security by using one way hash chains and nested keyed message authentication codes, as well as multipath routing. Yao *et al.* in [9] presented a multipath secure routing protocol for WSNs. However, their approach requires the acknowledgement between every pair of nodes in routing. Therefore, it will cause large overhead to the data collection of the WSN.

Compared with the existing works in this field, our approach use a novel tracing-feedback mechanism, which makes full use of the routing functionality of WSNs to improve the quality of data collection. The secure paths here are potentially safe for data collection. Therefore, routing via secure paths is much more secure than random multipath routing. The major difference between our approach and the existing multipath methods is that the process of constructing secure paths causes little overhead to sensor nodes and the algorithms are easy to implement and perform in resource-constrained WSNs.

## 3. Multipath Data Collection

Multipath routing has been used for different purposes in WSNs, such as load balancing, energy efficiency [10,11], *etc.* In this paper, we make use of multipath routing for secure data collection. We use a (*t*, *n*)-threshold secret sharing algorithm, e.g., Shamir’s algorithm [12], to encode a sensed data packet. When a sensor node wants to send a packet to a destination node (often the sink), it first breaks the packet into *N* shares according to the secret sharing algorithm. Each share is then transmitted to some randomly picked neighbor. The (*t*, *n*)-threshold secret sharing algorithm is illustrated as follows:

1. Without loss of generality, we assume that the data is denoted by a number *D*. We choose a number *p* larger than both *D* and *n*.
2. We generate a collection of coefficients *a*_1_, *a*_2_, …, *a_t_*_−1_ from [0, *p*) and construct a *t* − 1 degree polynomial *q*(x) = *a*_0_ + *a*_l_*x* + ... + *a_t_*_−1_*x^t^*^−1^, in which *a*_0_ = *D*.
3. We evaluate *D*_1_ = *q*(1), *D*_2_ = *q*(2), ..., *D*_n_ = *q*(n). Here *D_i_* denotes *i^th^* share of the data.
4. We deliver different shares to different objects.
5. Given any subset of *t* of these *D_i_* values, we can calculate the coefficients of *q*(x) by interpolation, and then evaluate *D* = *q*(0). However, *t* − 1 (or less) of these values does not suffice in order to calculate *D*.

**Figure 1.**
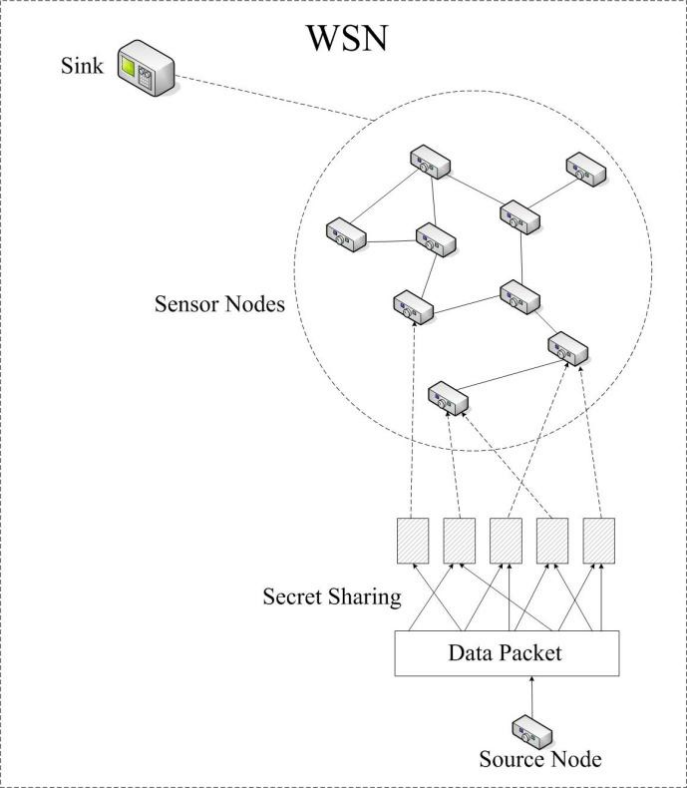
A WSN with secret sharing mechanism for data collection.

Therefore, we can break a data packet into a collection of shares by using the (*t*, *n*)-threshold secret sharing algorithm and deliver different shares via different routing path (see Figure 1). We can extend existing multipath routing algorithms like AOMDV [13], SMR [14] to achieve secure routing in WSNs. Moreover, we intend to find as many routing paths as possible for a source node rather than using a set of disjoint paths like in AOMDV and SMR. We extend the algorithm given in [3] to generate routing path randomly for data collection. A data packet is broken into shares according to the (*t*, *n*)-threshold secret sharing algorithm and shares are transmitted to the sink via different paths. The algorithm in [3] does not consider the density of the sensor nodes in a WSN. If the degree or the number of neighbors of a node is small, there may be not enough candidates for delivering shares. Moreover, different nodes in a WSN have different degree values, a fixed (*t*, *n*)-threshold cannot satisfy every node in the WSN. We extend the algorithm in [3] with an adaptive (*t*, *n*)-threshold that varies according to the degree of node.

The adaptive data collection (ADC) algorithm is illustrated as follows:

1. To a source node *S* that intends to send a data packet *D*, if its degree is larger than a threshold value *k*, we set *n* to the degree of the node, which is the number of the neighbors of the node. Moreover, we set *t* to a number that is less than *n*. Otherwise, the node sends *D* by normal routing until *D* reaches a node with enough degree.
2. We break *D* into *n* shares according to the (*t*, *n*)-threshold secret sharing algorithm.
3. For each share, we perform node selection using one of the four distributed random propagation mechanisms in [3].
4. In this way, the shares of *D* are forwarded by a collection of relay nodes until they reach the sink.

**Figure 2.**
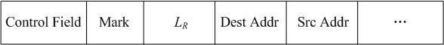
The structure of the data packet.

In the algorithm above, we consider the degree of sensor nodes. If the degree of a sensor node is small, it is not necessary to break a data packet into shares. We use an adaptive mechanism to control process of breaking data packet into shares. We will forward a data packet until it reaches a node that has a degree value large enough for (*t*, *n*)-threshold algorithm. In order to distinguish data shares and the original data packet, we should add an additional flag in the beginning of the data packet (see Figure 2). The data packet also contains a routing sequence field *L_R_* (used later).

## 4. Secure Path Algorithm

In this paper, we consider a relatively simple WSN model. Each sensor node in the WSN is battery-powered and has limited sensing, computation and wireless communication capabilities. The sink is a data collection center equipped with sufficient computation and storage capabilities. Sensor nodes generate sensed information and aggregate data packets. The sink collects data from sensor nodes periodically. The routing layer of WSNs is threatened by various attacks. However, due to the focus of this paper, it will not be further discussed and here we consider only attacks of packet dropping like selective forwarding and black hole. We assume that compromised nodes, in order to allay suspicions, selectively drop only a small proportion of all packets passing by rather than every packet. If a compromised node drops every packet, it is a black hole, which can also be handled by our approach.

The process of data collection in WSN is a relay of data packets from the source node to the sink. The approach in this manuscript is mainly based on an assumption that **if a data packet from the source successfully arrives at the sink, the path from the source to the sink is more likely to be safe for subsequent data collection**. Therefore, we can make use of such historical information about data collection to improve the quality of the data collection and even perform intrusion detection.

### 4.1. Feedback-Based Secure Path Construction

**Figure 3.**
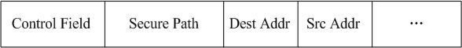
The structure of the notification packet.

**Figure 4.**
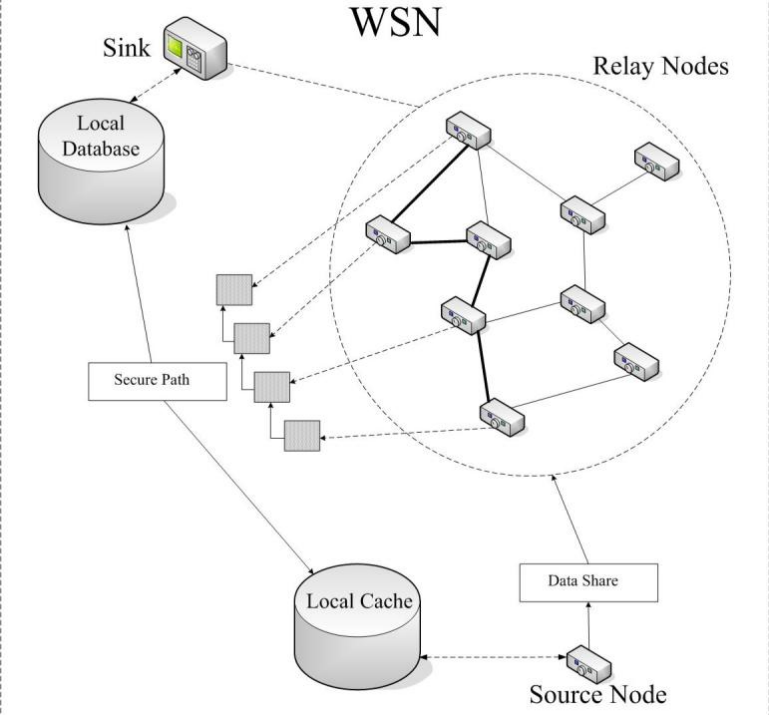
An illustration of the FSPC algorithm for secure data collection.

Here we propose a feedback-based secure path construction (FSPC) algorithm to support secure data collection. We try to use a *tracing/feedback mechanism* for this purpose. The algorithm is illustrated as follows:

1. A source node *S* sends a data packet according to the ADC algorithm. To each share of data packet, *S* attaches an identity list *L_R_* to it. Initially, *L_R_* is empty.
2. When a sensor node *S_k_* receives a share, if it is a normal node, it adds its identity *d_k_* to *L_R_* (it is possible that compromised nodes also do so in order to disguise themselves).
3. On the arrival of the share, the sink extracts *L_R_* = {*d*_1_, *d*_2_, …, *d_n_*} (*d_i_* refers to the identity of the node *S_i_*) from the share and stores it in its local database. Here *L_R_* is called a *secure path* in this case.
4. The sink adds *L_R_* to a notification packet (see figure 3) and sends the packet to *S* according to *L_R_*. The notification packet contains the secure path for data collection.
5. When a sensor node *S_j_* receives the packet, if its identity *d_j_* is within *L_R_*, it extracts a sub-path *P_j_* = {*d_j_*_+1_, *d_j_*_+2_, …, *d*_n_} from *L_R_* and stores it into its local cache. *S_j_* extracts its next-hop node *S_j_*_−1_ with identity *d_j_*_−1_ from *L_R_* and forwards the packet to it. *P_j_* is also called a sub-path of the secure path *L_R_* for *S_j_*.
6. On the arrival of the packet, *S* extracts *L_R_* from the packet, and stores it in its local cache (see figure 4).

In this algorithm, each normal sensor node in a routing path adds its unique identity to the data packet. When the data packet reaches the sink, it involves a routing path that consists of a list of the identities of normal sensor nodes. It means that the path is potentially safe for data collection and can be used again by the source node in the future. A complete secure path is always terminated and collected by the sink. Here we use a *feedback mechanism* to notify the source node that requires the path for future data collection. The sink sends back a notification packet that contains a secure path to the source node. The task of notification is at intervals rather than immediately in order to reduce the overhead of the WSN. We can formally represent a secure path as a triple <*S*, *L_R_*, *C*>, where *S* is the source node for the path, *L_R_* is the identity list and *C* is a counter with an initial value λ (λ > 0). The value of *C* denotes the trust value for a secure path and a path with larger counter value is safer.

**Figure 5.**
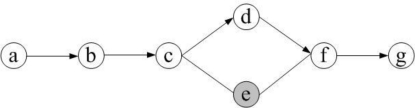
A simple example for secure path in WSN.

The secure path *L_R_* from the source node *S* to the sink is a complete path for data collection. Therefore, it is called a *global secure path*. The secure path *P_j_* from an intermediate node to the sink is a part of the global secure path, so it is called *local secure path*. Local secure paths can be extracted from a global path. With FSPC, we can get one global secure path as well as a collection of local secure paths. For example, a source node *a* wants to send data to the destination node *g* (see figure 5). Assume node *e* is a compromised node. Then the path {*b*, *c*, *d*, *f*, *g*} is a complete secure path. Paths like {*d*, *f*, *g*} are local secure paths.

Here the notion of secure path does not mean that the path is safe for data collection. A secure path may include compromised nodes. This is mainly because that a compromised node drops a data packet with a probability. If a compromised node does not drop any data packet during the process of secure path construction, it will be considered potentially safe and be included in the path. In the worst case, each compromised node does not drop the data packet on the stage of secure path construction, in order to be involved in a secure path. Then each compromised node will appear on a secure path, which leads to a very low success ratio of data transmission. Therefore, when we say a path is a secure path, it only means that the path is currently safe for data collection.

### 4.2. Secure-Path based Data Collection

As long as a source node receives enough secure paths from the sink, it is able to send data via these paths. Therefore we can improve the ADC algorithm in Section 3 by using secure paths. The secure-path based data collection (SPDC) algorithm is illustrated as follows:

(1) When a source node *S* intends to send a data share to the sink, it first checks its local cache. If there are secure paths, it selects a secure path *P* = <*S*, *L_R_*, *C*> with the largest counter value from its local data repository. *S* adds *L_R_* = {*d*_1_, *d*_2_,…, *d_n_*} to the beginning of the data share. If there are no secure paths in the local cache of the relay node, it just performs random multipath routing as the ADC algorithm in Section 3. If *S* has no secure paths at all, it performs path construction as the FSPC algorithm in Section 4.1.
(2) Before sending the share, *S* first checks whether the node *S*_1_ with identity *d*_1_ is in its neighbor list. If the node is not in the list, it just performs random multipath routing. Otherwise, it sends the share to the node *S*_1_ with *d*_1_.
(3) When a sensor node *S_k_* receives a share, it first checks whether there is any secure path in the head of the share. If not, it performs random multipath routing and path construction. Otherwise, it checks whether the node *S_k_* _+ 1_ with identity *d_k_* _+ 1_ is in its neighbor list. If the node is not the list, it just performs random multipath routing. Otherwise, it sends the share to the node *S_k_* _+ 1_ with *d_k_* _+ 1_.

*If the share reaches the sink successfully:*

(4) On the arrival of the share, if there is a secure path in the share, it means every relay node has used the path and the sink just sends back an empty notification to *S*. Otherwise, the sink extracts the identity list as a new secure path from the share, updates its local database, and sends back a notification with the newly-constructed secure path to *S*.
(5) The relay nodes on the path update their local cache with sub-paths.
(6) On the arrival of the notification, *S* extracts new secure path (if any) from the packet, and stores it in its local cache.

*If the share is dropped or does not reaches the sink within the time span allowed:*

(7) *S* does not receive a notification from the sink, and then it just decreases the counter of *P* by 1.
(8) If the counter of a secure path is cleared, *S* will remove it from its local cache. *S* will resend the share if possible.

From this algorithm, we can see that a secure path is not considered secure all the time. Secure paths are evaluated by their quality of service (QoS) for data collection. The SPDC algorithm deals with selective forwarding attacks by using a scoring mechanism. In this way, we can exclude as many compromised nodes as possible from data collection. The local cache for secure paths of a source node therefore changes dynamically to support more secure data collection in WSN.

## 5. Simulation

In this section, we construct a simulation to evaluate the performance of the proposed approach. The major metric for performance evaluation is the packet interception probability (PIP) for a source node, defined as the ratio of the number of intercepted data packets to the total number of packets from the source node. To better understand the capability of the randomized multi-path routing algorithms in bypassing black holes, we also compare the performance of our approach with the original algorithms in [3]. The basic setting for the simulation is illustrated in Table 1. Here the parameter drop rate refers to the probability that a compromised node drops a data packet.

### 5.1. Packet Interception Probability Evaluation

**Figure 6.**
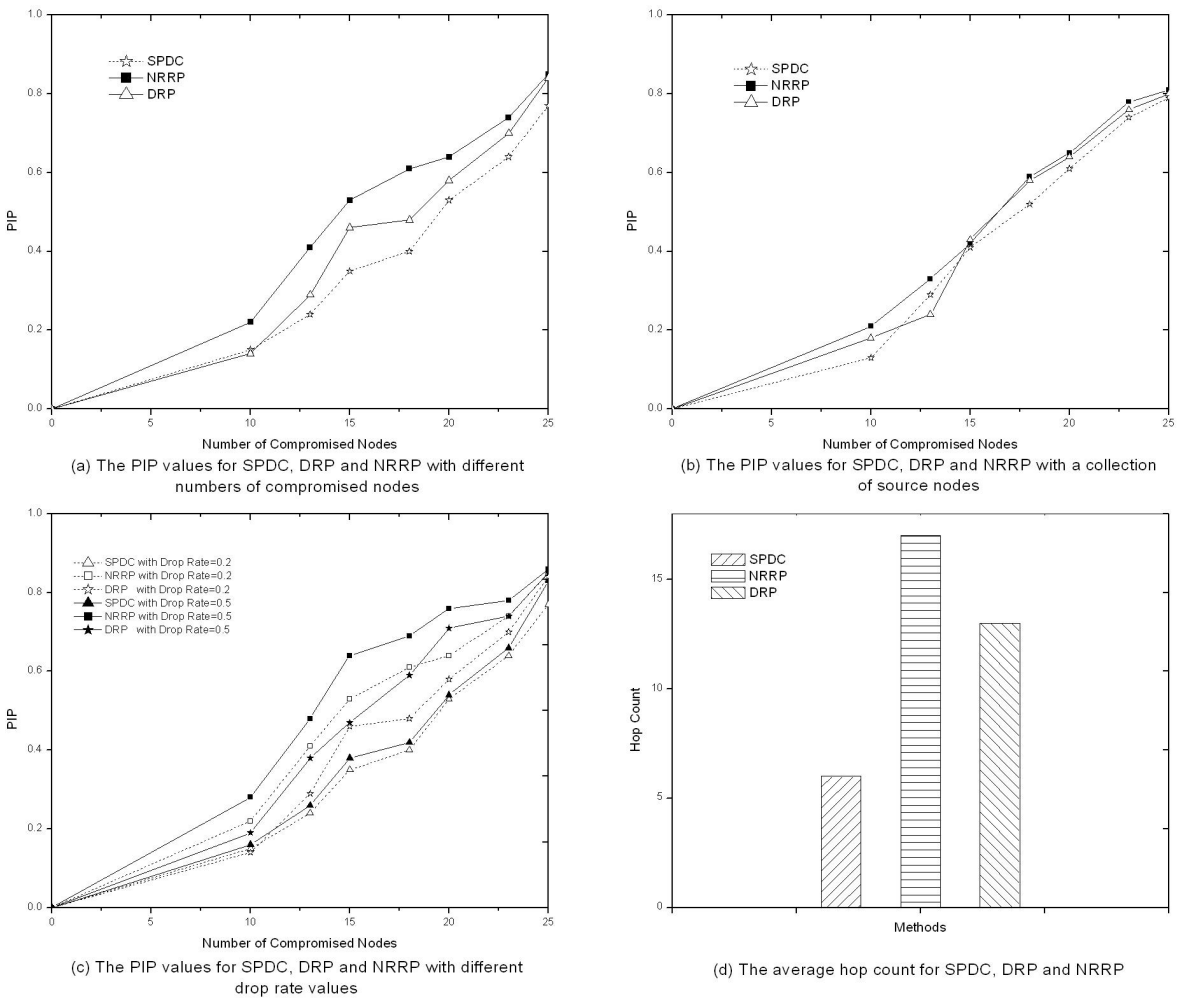
The simulation results for the performance evaluation of SPDC.

We first fix the location of a source node that sends data to the sink. We first investigate the PIP for the source node under different numbers of compromised nodes. To each number of compromised nodes, we evaluate the average PIP for the source node. Figure 6(a) shows a plot of the PIP for the source node under different numbers of compromised nodes. It is obvious to see that the PIP increases when the number of compromised nodes becomes larger. When half of the sensor nodes are compromised nodes, most of the data packets are intercepted. We also compare the performance of the SPDC algorithm (including ADC and FSPC) with that of NRRP algorithm proposed in [3]. As can be seen in the figure, the performance of SPDC is better than NRRP with the same number of compromised nodes. When the number of compromised nodes is very small or large, the performance of the two algorithms is very close. However, SPDC behaves much better than NRRP with the number of compromised nodes falling into the extent (13, 18).

**Figure 7.**
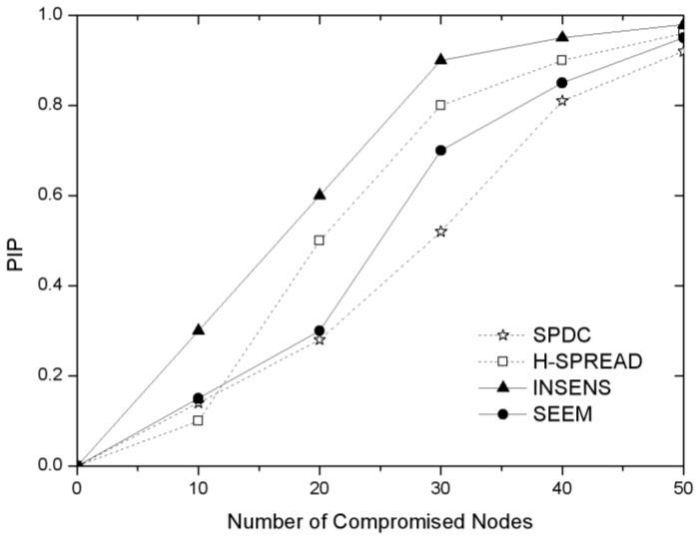
The performance of SPDC against H-SPREAD, SEEM and INSENS.

Moreover, we also compare the performance of SPDC with H-SPREAD [5], SEEM [7] and INSENS [8]. We change the number of the sensor nodes to 100 and evaluate PIP under different numbers of compromised nodes (see Figure 7). Here H-SPREAD is implemented with T = 9, and M = 3. INSENS is implemented with two paths, and one base station. It can be seen from the figure that the over performance of SPDC is better than the other three approaches. SPDC behaves much better than the other approaches with the number of compromised nodes falling into the extent (20, 40).

### 5.2. Performance Evaluation with Source Node Set

The work in [3] performs simulation by using a fixed source node that sends data to the sink. Using only a fixed source node is not enough to simulate the behaviors of WSNs. In practice, data is always generated by different sensor nodes distributed across an area. It is insufficient to evaluate the PIP with a fixed source node. In fact, our approach can achieve better performance as long as there is secure path from the source node to the sink. Therefore, we evaluate the overall PIP with a collection of source nodes. We select a collection of source nodes and each one is likely to generate data and send it to the sink. Then we evaluate the overall performance for our approach for the collection of source nodes.

The process of simulation with a collection of source nodes is similar with that of a fixed source node. We evaluate the data collection algorithms for each source node in the collection and record the accumulative result for the collection. Here the number of the source nodes in the collection is 10 (see Table 1). As can be seen in Figure 6(b), the performance of SPDC is better than DRP and NRRP with the same number of compromised nodes. However, the advantage of SPDC is not very obvious against the other algorithms in this situation. The performance of DRP and NRRP is close to SPDC.

### 5.3. Performance Evaluation under Different Drop Ratios

We change the drop rate of compromised nodes to different values and evaluate the performance of the approach under different drop rate values. A larger drop rate implies that more data packets will be dropped by compromised nodes. Therefore, the PIP is more likely to increase when the drop rate value increases. As illustrated in Figure 6(c), both SPDC and NRRP achieve a higher PIP value when we change the drop rate from 0.2 to 0.5.

It can be seen that the performance of SPDC is better than NRRP when the drop rate value is large. The performance of SPDC at drop rate of 0.5 is close to that at 0.2. In the contrast, the overall PIP of NRRP increases a lot when the drop rate changes from 0.2 to 0.5. It means that the performance of NRRP is poor when the drop rate is high. With a large drop rate value, it is easier for SPDC to exclude compromised nodes from secure paths in the beginning, which results in a better performance. However, to NRRP, a large drop rate value just increase the number of data packets being dropped. Therefore, SPDC performs better than NRRP when the drop rate is high.

### 5.4. Hop Count Evaluation

We also evaluate the average number of hops of the end-to-end route generated by the approach. We fix the number of compromised nodes to 10 and evaluate the average hop count for each algorithm with the same number of data collection tasks. Figure 6(d) depicts a plot of the average hop count for the data collection with different algorithms. It can be seen that the overall performance of SPDC is better than DRP and NRRP. It takes fewer hops by using SPDC to perform data collection.

As long as a secure path is constructed, subsequent data packets will be sent through the path. It is no need to perform random routing any more. The SPDC algorithm reduces additional random data packet forwarding in random dispersive algorithms like DRP and NRRP. Therefore, the hop count of data collection with SPDC is much fewer than that with DRP or NRRP.

### 5.5. Transmission Delay Evaluation

Although we can enhance the security of data collection in a WSN by using methods like SPDC, the performance of the WSN is affected because of additional communication and storage overhead. The SPDC algorithm affects the performance of data collection in WSNs. A secure routing mechanism will affect the performance of a WSN in different aspects like transmission delay, energy consumption, load balance, *etc.* In this manuscript, we focus on the impact of the SPDC algorithm on the transmission delay of WSN. We evaluate the performance of the algorithm by several groups of parameters. Table 2 shows the performance of SPDC under different groups of parameters. It can be seen that the overall delay of data transmission is higher when the number of the nodes in the WSN is larger. It is mainly because that the hop count increases when the network scale becomes large. A large hop count results in high transmission delay. We also note that the overall delay decreases as the times of transmission decrease under a fixed group of parameters. It is mainly because that the delay caused by routing path discovery and secure path construction is fixed and independent of the times of transmission. Therefore, the average delay decreases when the times of transmission increase.

## 6. Conclusions

In this paper, we propose a novel approach of secure data collection for WSNs. We explore secret sharing and multipath routing to achieve secure data collection in a WSN with compromised nodes. The key component of the approach is a novel tracing-feedback mechanism, which makes full use of the routing functionality of WSNs to improve the quality of data collection. The advantage of the approach is that secure paths are constructed as a by-product of data collection and are potentially safe for subsequent data collection. The process of constructing secure path causes little overhead to the sensor nodes in a WSN, while perform routing via secure paths is more secure than random multipath routing. Compared with the existing works in this field, the algorithms of the approach are lightweight to the resource-constrained sensor nodes in WSNa. According to the simulation results, the performance of the proposed approach is better than the other approaches with a similar purpose. In all, our work tries to take a step forward secure data collection for WSNs.

Future works may include: (1) improving the efficiency of the algorithms to reduce the overhead of secure path notification; (2) making use of the secure paths in a local cache or database to detect compromised nodes and perform intrusion detection for WSNs; (3) considering a more complex WSN model to implement and evaluate the approach.

## Figures and Tables

**Table 1.** The major parameters for the simulation.

**Parameter**	**Value**
Number of Sensor Nodes	50
Threshold Value *k*	5
Drop Rate	0.2
Initial Counter Value λ	3
Number of Source Nodes in a Collection	10

**Table 2.** The transmission delay of SPDC under different groups of parameters.

**Number of Sensor Nodes**	**Number of Compromised Nodes**		**Transmission Times**	**Average Delay (assume one-hop delay is 1)**
50	10	500		37
50	10	1,000		35
100	20	500		52
100	20	1,000		47
